# Aesthetic appreciation of musical intervals enhances behavioural and neurophysiological indexes of attentional engagement and motor inhibition

**DOI:** 10.1038/s41598-019-55131-9

**Published:** 2019-12-06

**Authors:** P. Sarasso, I. Ronga, A. Pistis, E. Forte, F. Garbarini, R. Ricci, M. Neppi-Modona

**Affiliations:** 1 SAMBA (SpAtial, Motor & Bodily Awareness) Research Group, Department of Psychology, University of Turin, Turin, Italy; 2 MANIBUS Lab, Department of Psychology, University of Turin, Turin, Italy

**Keywords:** Emotion, Learning and memory, Human behaviour

## Abstract

From Kant to current perspectives in neuroaesthetics, the experience of beauty has been described as *disinterested*, i.e. focusing on the stimulus perceptual features while neglecting self-referred concerns. At a neurophysiological level, some indirect evidence suggests that *disinterested aesthetic appreciation* might be associated with attentional enhancement and inhibition of motor behaviour. To test this hypothesis, we performed three auditory-evoked potential experiments, employing consonant and dissonant two-note musical intervals. Twenty-two volunteers judged the beauty of intervals (Aesthetic Judgement task) or responded to them as fast as possible (Detection task). In a third Go-NoGo task, a different group of twenty-two participants had to refrain from responding when hearing intervals. Individual aesthetic judgements positively correlated with response times in the Detection task, with slower motor responses for more appreciated intervals. Electrophysiological indexes of attentional engagement (N1/P2) and motor inhibition (N2/P3) were enhanced for more appreciated intervals. These findings represent the first experimental evidence confirming the *disinterested interest hypothesis* and may have important applications in research areas studying the effects of stimulus features on learning and motor behaviour.

## Introduction

Most current theories of aesthetics describe aesthetic appreciation as a mental state focusing on the stimulus perceptual features, while neglecting self-referred concerns^1–8^. This idea of aesthetic pleasure as *disinterested*, originated in the western positivist philosophical tradition. Kant, in the ‘Critique of Judgement’, defined taste as “the faculty of judging an object […] by an entirely disinterested satisfaction or dissatisfaction”^9^. This notion was further adapted into a psychological theory of aesthetics by the philosopher Schopenhauer^10^, according to whom aesthetic experiences free the observer from “will”, allowing him or her to achieve a transitory will-less [willenlos] perception of the world.” Therefore, aesthetic appreciation is defined as independent from any material or social reward or loss (i.e., *disinterested*; for a recent discussion see e.g., Kreitman^11^) and at the same time prompted by a special attitude of attention (i.e., focused on the stimulus features; see e.g., Stolnitz^12^). For the philosopher Dewey, aesthetic experiences involve an intense engagement in the ever-changing present moment and stand out from more mechanical and routine interactions with the environment^13^. The temporary suspension of prototypical responses that results from psychological distance (i.e. absence of personal goals or threats) makes room for a higher intensity of the felt sensations and emotions elicited by beautiful objects (Distancing-Embracing model^8^). This enables observers to fully embrace the “here and now” of perception for its own sake, and the subjectively felt intensity of sensations being rewarding in its own right^14^.

Interestingly, recent neuroaesthetic research has proposed neurofunctional models of aesthetic appreciation that refer to the same theoretical framework described above. Aesthetic pleasure is considered as a peculiar reward, directed to promote contemplation (i.e., “sensing and learning pleasures”^5,15–18^), while preventing the craving for objects by inhibiting motor activation^3^. We will refer to this link between aesthetic appreciation, attention to stimulus features and inhibition of motor behaviour, as the *disinterested interest hypothesis*.

Some neuroimaging results support this hypothesis. Through an electrophysiological study, de Tommaso *et al*.^19^ found increased motor inhibition in response to beautiful images as compared to ugly ones. More specifically, the amplitude of the event-related potential (ERP) P3 component, known to be modulated by motor-inhibition, was greater for visual stimuli perceived as more beautiful than for neutral or ugly images. Kawabata and Zeki^20^ found significantly greater fMRI activations in bilateral motor cortices during the observation of paintings judged as ugly, as compared to paintings rated as beautiful. Interestingly, motor activations were linearly increasing with the subjectively perceived stimulus ugliness. Similarly, Di Dio and colleagues found increased activations peaking in the left motor cortex after the presentation of images of statues rated as ugly compared to beautiful images^21^. Moreover, in the auditory domain, the existence of a relation between motor responses to sounds and their pleasantness has also been described^18^. Roy and colleagues^22^ found that the startle eye blink reaction amplitude was larger during unpleasant compared with pleasant consonant intervals. Moreover, a number of neuroimaging studies^16,23–27^ revealed the presence of enhanced sensory processing for more appreciated visual and auditory stimuli, which might be attributed to the effect of increased attentional engagement^28–30^. Additionally, fMRI studies investigating “disinterested” aesthetic judgements showed partially functionally dissociable networks underlying judgements of beauty and more pragmatic (e.g. symmetry) judgments^25^. During aesthetic judgements only, more appreciated visual stimuli caused a “beauty-induced” signal boost in higher visual processing areas^25^ that mimics the effect of increased attention. However, to the best of our knowledge, there is no direct evidence of a link between beauty-related motor inhibition and attention engagement towards beautiful stimuli, as postulated by the *disinterested interest hypothesis*.

In the present study, we aim at testing this hypothesis, both at a behavioural and at an electrophysiological level, with auditory stimuli. Sounds, as well as images, can induce an aesthetic response, and, at the same time, it is possible to control for sounds’ basic features (e.g., frequency, duration, complexity, volume) in a very precise way. Previous studies demonstrated that ERPs may provide neurophysiological indexes of both attentional engagement/selection^31,32^ and motor inhibition^33–35^, thus making EEG a suitable technique to investigate the possible correlates of disinterested aesthetic appreciation. We will present content-free auditory stimuli, consisting in more or less consonant two-note intervals (i.e., two synthetic tones displayed simultaneously), because consonance is known to influence aesthetic appreciation^36^ and to modulate cortical responses measured with EEG^37–41^. Even though some studies found peference for mild dissonance over consonance^42^, more consonant musical intervals are normally (especially in non musicians^43^) more appreciated than dissonant ones^36,44–48^. Importantly, it is possible to produce consonant and dissonant intervals sharing comparable physical features by just varying the ratio between the frequency (Hz) of single tones^36^ (§ Stimuli). This was crucial in the present experiment to control for potential confounding effects, due to tones’ basic physical features, known to affect EEG responses^49^.

To test our hypothesis, we performed three EEG experiments. In *Experiment 1*, participants were asked to evaluate the beauty of single intervals (aesthetic judgement task, from now on *AJ* task). In *Experiment* 2 participants listened to the same intervals intermixed with white noise (intervals were presented in 50% of trials) and had to respond as fast as possible by pressing a button whenever they heard an interval (*Detection* task). This task aimed at investigating the relationship between aesthetic experience and motor behaviour. In *Experiment 3*, participants had to perform a *Go-NoGo* task, which is usually employed to investigate motor-inhibition mechanisms and their electrophysiological correlates^50,51^. In this task subjects had to respond to frequent *Go* stimuli, while avoiding to respond to infrequent *NoGo* stimuli.

If the *disinterest interest hypothesis* is correct, we expect the following results: 1) Slower response times in the detection task for more appreciated intervals (as a behavioural index of related motor inhibition); 2) The enhancement of ERP components related to motor inhibition (the N2/P3 complex) for more appreciated intervals; 3) The enhancement of attention-related ERP components (such as the N1/P2 complex) for more appreciated intervals.

## Results

### Experiment 1 (AJ task)

#### Behavioural results

**Figure 1:**
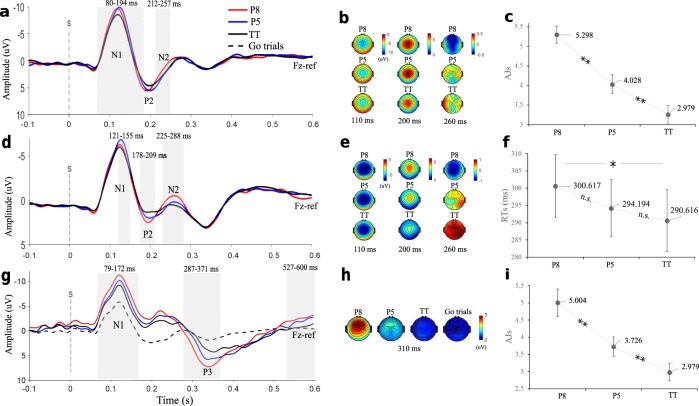
AEPs and behavioural results. Panel a shows grand-average AEPs recorded at Fz during the *Aesthetic Judgement* task. AEPs elicited by different interval types during the *Detection* and *Go-Nogo* task are represented in panel d and g, respectively. Shaded areas represent significant time-clusters evidenced by the point-by-point ANOVA. Scalp-maps depict voltage distribution registered during the *Aesthetic Judgement* (panel b) and *Detection* task (panel e) at 110 ms (N1), 200 ms (P2), 260 ms (N2). Panel h shows voltages registered on the scalp at 310 ms post-onset (P3) during the *Go-NoGo* task. Panels c, f and i depict all subjects’ mean AJs from *Experiment 1*, RTs from *Experiment 2* and AJs from *Experiment 3*, respectively. Bars represent standard errors. Asterisks represent significant differences in single subjects’ mean AJs and RTs between different interval types evidenced by two-tailed t-tests (*p < 0.05, **p < 0.001, n.s = not significant). P8 = perfect octaves, P5 = perfect fifth intervals, TT = tritone intervals.

Aesthetic judgements (AJs) were significantly modulated by consonance (*F* = 45.682, *p* < 0.001, *η*^2^_*p*_ = 0.685, observed-power = 1): more consonant intervals were, on average, more appreciated than more dissonant ones on a 1–9 Likert scale (5.298 for Octaves, 4.028 for Fifths and 3.26 for Tritones, Fig. 1c).

Auditory evoked potential (AEP) results: The point-by-point ANOVA (corrected with 1000 permutations) highlighted two significant clusters with a fronto-central distribution. On Fz, the main effect of ‘Condition’ was a significant source of variance within the time window of 80–194 ms and 212–257 ms, corresponding to the latency of the N1/P2 complex and the N2 component, respectively (Fig. 1). Post-hoc pairwise comparisons (cluster corrected point-by-point t-tests comparing waveforms corresponding to the three different interval types) were performed given the significant effect of “Interval type”. The results of point-by-point t-tests are fully reported in Table 1.

**Table 1:** Post-hoc point-by-point t-tests.

(I) Interval type	(J) Interval type	Significant clusters latencies (ms post-onset)
*Experiment 1*		
P8	P5	126–174
		207–259
P5	TT	78–135
		160–193
		229–258
	TT	115–156
*Experiment 2*		
P8	P5	126–158
		222–281
	TT	177–207
		235–291
P5	TT	115–149
		185–213
*Experiment 3*		
P8	P5	40–75
		113–165
	TT	82–186
		274–370
		519–601
P5	TT	544–623

This table reports the latencies of significant clusters evidenced at Fz by the point-by-point t-tests comparing waveforms registered during different interval types (I vs. J).

P8 = Octaves; P5 = Fifths; TT = Tritones.

**Figure 2:**
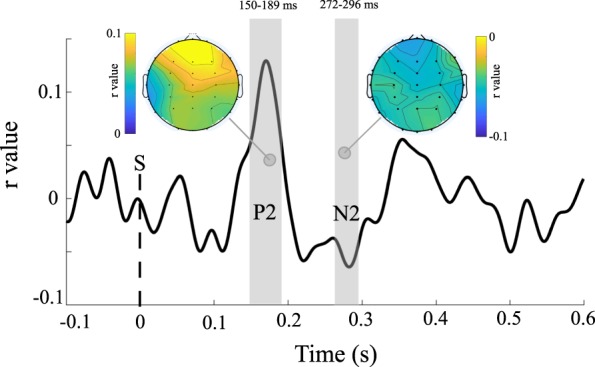
Point-by-point trial-by-trial correlation analysis. The graph shows mean (averaged across all participants) correlation coefficients between single subjects’AJs (*Experiment 1*) and signal amplitudes at channel Fpz. Shaded areas represent significant clusters evidenced by the point-by-point t-test comparing the 22 single subjects’ r values against 0. The t-test on r values between amplitudes and AJs revealed two significant clusters (150–189 and 272–296 ms) corresponding to P2 and N2. Scalpmaps represent the distribution of mean r values at 180 and 280 ms post-onset.

Mean correlation coefficients (averaged across participants) between amplitudes at channel Fpz and AJs are depicted in Fig. 2. The point-by-point t-test on single subjects’ r values highlighted a significant positive correlation between trial-by-trial P2 amplitudes and AJs, as revealed by the presence of a significant cluster centred at the latency corresponding to the peak of the P2 component (150–189 ms). Moreover, a second significant time-cluster at 272–296 ms revealed a negative correlation between N2 amplitudes and AJs. As shown by scalpmaps in Fig. 2, r-values were peaking at Fpz at 180 and 280 ms post-onset.

### Experiment 2 (Detection task)

#### Behavioural results

Omission rate was 0% in the *Detection* task. On average, 8 responses per participant (2.85% of the total) were considered as outliers (i.e. RTs exceeded two standard deviations from the single subject’s mean) and were excluded from subsequent analyses. Outliers were equally distributed across interval types.

**Figure 3:**
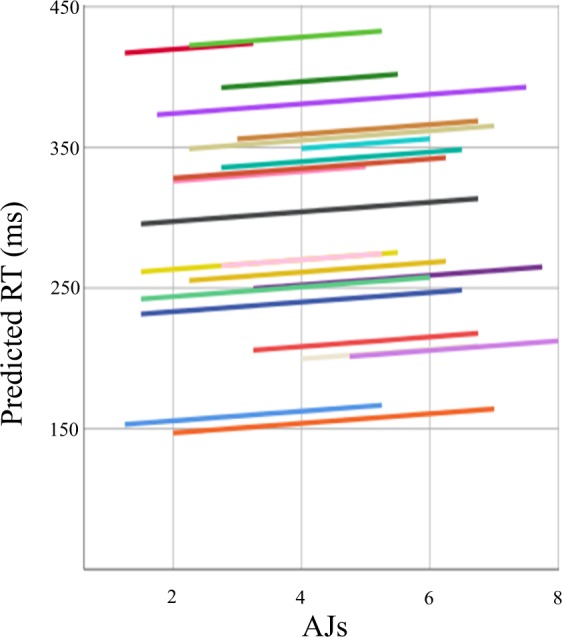
Linear mixed-model. The 22 coloured lines represent single participants’ predicted RTs, based on the parameters estimated by the mixed-model analysis (§ Data analysis, Behavioural data) and observed AJs. Predicted RTs were defined as a function of subjects’ ID and observed AJ. The positive slope of the lines indicates a positive statistically significant (§ Results, Experiment 2) relation between AJs and RTs.

The repeated measures ANOVA performed on RTs failed to reveal a significant effect of the factor interval type (*F* = 2.678, *p* = 0.08, *η*^*2*^_*p*_ = 0.113, observed-power = 0.502). Interestingly, however, RTs were on average slower for more appreciated intervals (300.617 ms for Octaves, 294.194 for Fifths and 290.616 for Tritones, Fig. 1f). Moreover, based on our hypothesis #1 and despite the fact that the main effect of interval type was not significant, we performed post-hoc analyses (two-tailed t-tests), to verify whether pair-wise comparisons between RTs belonging to different interval types yielded any significant result. Post-hoc comparisons revealed a significant difference between RTs to Octaves and Tritones (*t* = 2.066; *p* = 0.026; Cohen’s d = 0.075). Furthermore, results from the linear mixed-model analysis evidenced that AJs could significantly predict RTs (estimate of the effect = 3.133; 95% CI: 0.147 + 6.199; *p* = 0.04; *t* = 2.062). This result was significant after correction for multiple comparisons (Benjamini–Hochberg correction; false discovery rate: 10%; total number of tests in the study: 30). Crucially, as we expected, predicted RTs increased with AJs (see Fig. 3*)*.

#### AEP results

Similarly to *Experiment 1*, the point-by-point ANOVA (corrected with 1000 permutations) evidenced three significant clusters, with a wide fronto-central distribution. On Fz, the main effect of ‘Interval type’ was a significant source of variance within three time windows: 121–155 ms, coinciding with the latency of N1; 178–209 ms, corresponding to the latency of the P2 component; and 225–288 ms, coinciding with the N2 component (Fig. 1d). Therefore, the point-by-point analysis revealed that N1, P2 and N2 amplitudes were all significantly modulated by interval type during both the *AJ* and the *Detection* task.

**Figure 4:**
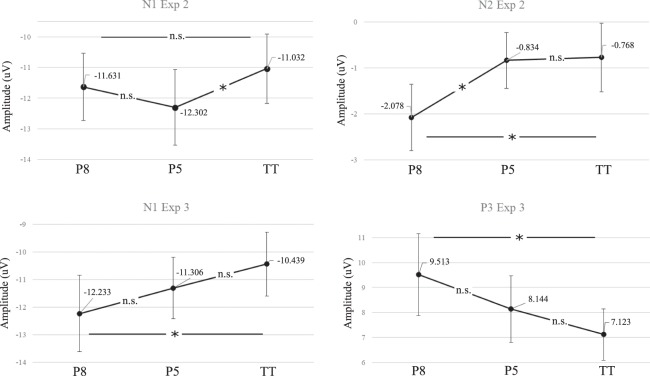
Mean peak amplitudes. The graphs show, for AEP components separately, all subjects’ mean peak amplitudes for the three interval types. Bars represent standard errors. Asterisks represent significant (p < 0.05) post-hoc pairwise comparisons between interval types (*p < 0.05, n.s = not significant). P8 = perfect octaves, P5 = perfect fifth intervals, TT = tritone intervals.

ANOVA results on peak amplitudes are fully reported in Table 2, where we also included pairwise comparisons between the levels of the factor *interval type* for components that were found to be significantly modulated by interval type. Results were corrected for multiple comparisons (Benjamini–Hochberg correction; false discovery rate: 10%; total number of tests in the study: 30). Overall peak analyses confirmed the findings highlighted by the point-by-point ANOVA, except for P2 peak in *Experiment* 2, where differences among interval types did not reach significance. In *Experiment 2*, N1 and N2 peak voltages were significantly modulated by interval type. Average peak amplitudes for significant components are represented in Fig. 4. With the exception of N1 in *Experiment 2*, average peak amplitudes generally showed the same trend as AJs, with greater peak voltages registered during the display of more appreciated consonant intervals.

**Table 2:** Peak amplitudes ANOVA.

ANOVAs on mean peak voltages						
Task	Component	Mean voltage at peak (µv)	F-value	p-value	Effect-size (Eta partial square)	Observed power
Detection-*Experiment 2*	N1	P8: −11.63; P5: −12.30; TT: −11.03	6.091*	**0.005**	0.225	0.865
	N2	P8: −2.07; P5: −0.83; TT: −0.77	6.609*	**0.003**	0.239	0.891
	P2	P8: 5.22; P5: 5.38; TT: 5.15	0.227	0.799	0.022	0.081
Go-NoGo-*Experiment 3*	N1	P8: −12.23; P5: −11.30; TT: −10.43	3.66*	**0.034**	0.148	0.643
	P3	P8: 9.51; P5: 8.14; TT: 7.12	3.85*	**0.029**	0.155	0.666
**Pairwise comparisons**						
**(II) Interval type**	**(J) Interval type**	**Mean difference (I-J)**	**St. err**.	**p-value**	**95% CI around Mean difference**	
**N1** ***Experiment 2***						
P8	P5	0.670	0.358	0.075	−0.073; 1.414	
	TT	−0.599	0.351	0.103	−1.329; 0.131	
P5	TT	−1.270^*^	0.382	**0.003**	0.475; 2.064	
**N2** ***Experiment 2***						
P8	P5	−1.244^*^	0.391	**0.004**	−2.058; −0.431	
	TT	−1.311^*^	0.440	**0.007**	−2.226; −0.396	
P5	TT	−0.066	0.385	0.865	−0.867; 0.735	
**N1** ***Experiment 3***						
P8	P5	−0.875	0.534	0.116	−1.987; 0.236	
	TT	−1.746^*^	0.791	**0.039**	**−**3.391; −0.101	
P5	TT	−0.870	0.582	0.149	−2.08; 0.339	
**P3** ***Experiment 3***						
P8	P5	1.316	0.828	0.127	−0.407; 3.039	
	TT	2.248^*^	0.870	**0.017**	0.438; 4.058	
P5	TT	0.932	0.738	0.221	−0.603; 2.467	

Amplitudes at peak latencies at Fz were extracted from single subjects’ mean ERP for relevant components in *Experiment 2* and *3*. Peaks voltages were entered in a one-way repeated measures ANOVA with Interval type as a within-subject factor. P-values highlighted in bold are significant after Benjamini–Hochberg correction for multiple comparisons.

P8 = Octaves; P5 = Fifths; TT = Tritones.

### Experiment 3 (Go-NoGo task)

#### Behavioural results

One participant was excluded from subsequent analyses for a technical problem which hampered the recordings. The remaining 21 participants of *Experiment* 3 performed, on average, 4.95 errors in the *Go-NoGo* task (incorrect NoGo trials were 5.893% of the total). Average error rates were comparable across interval types (6.247% for Octaves, 5.71% for Fifths and 5.71% for Tritones) and did not significantly differ between interval types as evidenced by a repeated measure ANOVA (*F* = 0.106, *p* = 0.9, *η*^*2*^_*p*_ = 0.005, observed-power = 0.065).

#### AEP results

Waveforms corresponding to the N1 (79–172 ms) and P3 (287–371 ms) components registered during the *Go-NoGo* task were significantly modulated by interval type (Fig. 1g), as evidenced by the point-by-point ANOVA (corrected with 1000 permutations). One additional significant time-cluster, centred around 550 ms post-onset, was evidenced by the ANOVA. This later cluster presumably corresponds to the negative rebound following the P3 oscillation. The ANOVA performed on peak voltages confirmed these results: N1 and P3 peak voltages were significantly modulated by interval type and were increased for more appreciated intervals (Table 2, Fig. 4).

## Discussion

In this study we aimed to test the *disinterested interest hypothesis* in the auditory domain, namely that aesthetic appreciation for more consonant two-note intervals is associated with attentional enhancement and motor inhibition. Based on this hypothesis we predicted, for more appreciated intervals: (1) Slower response times in the *Detection* task; (2) Significantly larger motor-inhibition AEP responses; (3) More pronounced AEP components related to attention enhancement. The results substantially confirmed our predictions. In *Experiment 1* and 3 we evidenced a subjective preference for more consonant intervals, thus replicating previous findings. Importantly, in *Experiment* 2, we showed that AJs predicted RTs in a simple detection task, as evidenced by the mixed-model analysis, with slower RTs for increasing AJs, thus confirming prediction #1. Moreover, results from *Experiments* 2 and 3 showed that attention and motor-inhibition related AEP components were significantly enhanced for more appreciated intervals, thus confirming predictions #2 and #3 (see also below). Overall, our behavioural and electrophysiological results seem to support the *disinterested interest hypothesis*. To our knowledge, this is the first empirical evidence of a direct link between aesthetic appreciation, attentional enhancement and motor-inhibition.

In the following paragraphs, we will discuss our electrophysiological results in relation to the existing literature, evidencing the possible evolutionary advantage of attentional enhancement and motor inhibition during aesthetic appreciation and the potential implications for basic and clinical research. We will discuss our results in the light of neuroimaging and behavioural results encompassing different sensory domains. This might be criticised, since some authors^52^ highlighted the need for domain-specific models of aesthetic judgements. It is possible that domain-specific models may be more appropriate to describe the processing of complex works of art^52^ (e.g. music pieces and paintings), which extends far beyond mere aesthetic pleasure and cannot be reduced to “core liking”^18^. Previous studies have demonstrated that, during the processing of works of art, domain specific processes normally apply to (low level) sensory processing, whereas domain general mechanisms apply to (higher level) central processing^52^. In our case, however, low-level perceptual correlates of “core liking”, triggered by basic stimuli (such as two-note intervals), might be predicted by domain-independent models as well. Indeed, neuroimaging and behavioural results in neuroaesthetics^28,30,53^ and neurocomputational models of aesthetic emtotions^54,55^ seem to suggest a common neurophysiological and behavioural pattern in the emergence of aesthetic appreciations across different sensory domains.

The N1/P2 complex amplitude has been frequently described as an index of attentional engagement^31,56–61^. In accordance with previous findings, in our study the N1/P2 complex amplitude was modulated by interval type in all three experiments, as evidenced by the point-by-point ANOVA, with larger amplitudes associated with preferred interval types. Interestingly, trial by-trial fluctuations in P2 voltages registered during *Experiment 1* significantly correlated with single trial AJs (*see* Fig. 2). Moreover, N1 peak voltages from *Experiment 3* were significantly larger for more appreciated intervals (see Fig. 4). Thus, overall, the point-by-point analyses on N1/P2 complex seem to indicate a significant enhancement of attentional-related responses for more appreciated interval types. Coherently, previous findings showed that expertise produced a similar effect: musical chords elicited larger P2 responses in professional musicians compared to laypersons, suggesting that experts develop specific abilities for music perception and cognition^62^.

It must be noticed, however, that peak analysis failed to find significant enhancement of N1/P2 complex for more appreciated intervals in *Experiment 2*, thus failing to replicate point-by-point results for what concerns N1 and P2. This apparently counterintuitive result might reflect the less ‘contemplative’ nature of the *Detection* task performed in *Experiment 2*, where participants had to respond to intervals as fast as possible. Task analysis, namely the comparison of contemplative vs. pragmatic responses to aesthetic stimuli, could inform us regarding the specific cognitive-affective processes underlying aesthetic judgement tasks^63–65^. This approach, however, might not be best suited to interpret our results because task demands and procedures among tasks are not directly comparable. Nevertheless, in the case of our study, more action-related neural resources were probably recruited in the *Detection* task: as a consequence, the expression of attentional-related components, was minimized (notice that N1 and P2 voltages were halved in the *Detection* task in respect to the *AJ* task and NoGo trials in the Go-NoGO task). Indeed, previous studies^63–65^ demonstrated that the amplitude of ERPs registered during the presentation of beautiful and ugly stimuli can be differently modulated when participants are judging stimulus beauty (i.e. contemplative condition) vs. more pragmatic aspects of the stimuli (i.e. non-contemplative condition). More pragmatic and action-finalized tasks (such as those requiring fast responses) seem to prevent the adoption of a contemplative attitude, typical of aesthetic judgements^1,12,66^, probably because motor preparation competes with perceptual mechanisms directing the attentional focus on stimulus features^8^. In other words, phenomenal and electrophysiological correlates of “core liking”^18^, i.e. embracing perception and sensations, might emerge only when perceivers are not goal-oriented (in the case of our study, when participants are not required to respond as fast as possible), thus allowing psychological distancing^8^.

In all our experiments, the amplitude of N2/P3 complex was systematically modulated by interval type, with larger amplitudes in response to more appreciated intervals. Crucially, N2/P3 amplitude enhancement is traditionally related to the recruitment of a “global suppression network”^67^ responsible for motor inhibition, and the slowing of motor output^68^. Apparently, N2 amplitude increases reflect early non-strictly motor aspects (i.e. cognitive) of inhibition^50^, finalized to overcome the usual stimulus-to-response mappings and to update the behaviour plan^69,70^. Therefore, N2 amplitude can be modulated even in tasks not directly requiring the inhibition of a motor response and, in such tasks, generally correlates with response times^71,72^. Consistently with this finding, we observed an increase of N2 voltages following the presentation of more appreciated intervals also in *Experiment 1* and *2*, where subjects were not required to inhibit their motor responses (i.e., they were not performing a Go-NoGo task). Notably, in Experiment 1, trial-by-trial fluctuations in N2 voltages were significantly correlated with AJs. P3 amplitude modulation, instead, has been traditionally related to later properly motoric stages of response inhibition^33,50,51^. As predicted by previous studies, we observed a significant P3 amplitude modulation only in Experiment 3 (the Go-NoGo task). Crucially, P3 average peak amplitudes registered during NoGo trials, were enhanced for more appreciated consonant intervals. Overall, the N2/P3 amplitude modulation observed in our experiments seem to indicate that aesthetic appreciation significantly fosters both non-motoric and strictly motoric stages of response inhibition, thus directly limiting motor activation. We propose that this inhibitory mechanism is finalized to support the contemplative “aesthetic attitude” (see also below).

Altogether, the enhancement of electrophysiological indexes of attentional engagement and motor inhibition might represent the neural counterpart of the neglect for self-referred concerns paired with an increment of attention for the stimulus perceptual features described by the theory of disinterest and distancing-embracing models of aesthetic experiences^8^ (see Introduction). However, it remains unclear what the evolutionary advantage of such a mechanism could be. Why do we divert attentional resources from action execution to the perception per se (i.e. contemplation) of more appreciated stimuli? This might be better understood within the theoretical framework of the free-energy principle^73–75^. Following the free-energy principle, agents select their action plans maximizing both expected utility (i.e. extrinsic value) and information gain or intrinsic epistemic value^76–78^. Since attention is a limited resource^79^, the most profitable strategy is probably to devote attentional resources, from time to time, either to maximize stimulus epistemic intrinsic value (i.e. updating and refining prior beliefs) or to maximize utilitarian extrinsic value based on prior beliefs^80–82^. But how does the nervous system choose where it should be most profitable to direct the attentional focus (toward perception *vs* toward action)? Previous theoretical models suggested that, in order to recognize stimuli which maximize epistemic value, intelligent systems (biological and artificial) have developed an intrinsic feedback on information gains (see Gottlieb *et al*.^82^ for a review). According to some authors^15,83–85^, the brain generates intrinsic rewards to stimuli with high informational content directly modulating the active sampling of sensory inputs. In accordance with this idea, we propose that aesthetic pleasure serves as an intrinsic reward in response to highly informative sensory interactions signaling to the nervous system the profitability of directing attention to present stimuli instead of modifying the environment through motor activation. This idea fits well with current models of aesthetic emotions, which posit that the intrinsic pleasantness of stimuli is of preeminent importance for their emergence^14^. Interestingly, previous research has already postulated the existence of a close link between aesthetic appreciation and stimulus informational value, with greater AJs for stimuli with the higher informational content^86–91^. Aesthetic pleasure has indeed been defined as a “meta-learning feedback”^92^ on successful perceptual learning dynamics^5,55,86,87,92^, i.e. when the cognitive system senses a progress in the refinement of mental representations and in the insightful^93^ creation of new ones^87,89,92^. Accordingly, the update of prior beliefs (which can be considered as an index of stimulus high informational value) was found to attract attention^94,95^ and to inhibit motor response^68,96^. Crucially, informational value per se also seems to trigger activations of midbrain reward-related areas^97^, which are usually found to correlate with aesthetic appreciation^20,98,99^. These data further support the presence of a direct link between aesthetic appreciation, stimulus information value, attentional enhancement and motor inhibition.

At first sight, the correlation between motor inhibition and aesthetic appreciation might seem at odds with other hypotheses which claim the active involvement of the mirror motor system in aesthetic appreciation, such as the “embodied simulation”^100–102^. According to this theory, the perception of beauty depends on the magnitude of empathic resonance with the content of works of art triggered by the activation of mirror neurons in motor^103,104^ and premotor^105^ areas. In our view, however, the two hypotheses are not mutually exclusive. Indeed, Gallese and colleagues argue that “embodied simulation” must be “liberated”, meaning that works of art (and the context in which they are perceived) must induce a potentiation of the mirroring mechanisms that are normally active in daily life^102^. According to Gallese, this potentiation is achieved via motor inhibition: “immobility, that is, a greater degree of motor inhibition, probably allows us to allocate more neural resources, intensifying the activation of bodily-formatted representations, and in so doing, making us adhere more intensely to what we are simulating”^102^ (p.48).

In our study, aesthetic preference, motor inhibition and attentional enhancement positively correlated with the consonance level of musical intervals. This result fits well with the hypothesis of a link between informational value and AJs, as discussed above. Aesthetic pleasure might be considered as a fundamental feedback to discriminate between fluently processed (i.e. informationally profitable) and noisy (i.e.“unlearnable”) signals. This argument might explain the preference for more consonant intervals given the evidence, well supported by behavioural and psychophysiological data, that consonant intervals are processed more fluently than dissonant intervals^37,46,106–109^. To this respect, it has been suggested that such processing efficiency enhancements might reflect the similarity between consonant intervals and conspecific vocalizations (which are mostly harmonic) to which the auditory system is tuned^36,44,110^.

In the present research, we propose that the experience of aesthetic appreciation might be considered as a cognitive state signalling to the system to refrain from acting in order to focus on present sensory stimulation to learn something new. Our results point to the possibility that the aesthetic value of stimuli can modulate cognitive functions, such as perceptual learning and memory retrieval (see also Lehmann & Seufert^111^ for a recent review), and future research should investigate this issue. Furthermore, the role of aesthetic emotions in automatically guiding attention toward perception and learning, instead of acting impulsively, is also potentially interesting for learning-oriented activities, such as teaching^112,113^, psychotherapy^114,115^ or communication more in general^13^. Moreover, the automatic attentional capture induced by aesthetic appreciation might be exploited in the design of experimental paradigms, where the attentional engagement of participants is crucial. Finally, the use of aesthetically more valuable stimuli might contribute to develop more effective neuropsychological rehabilitative protocols, for example with patients affected by mild cognitive impairments or dementia which manifest attentional and motivational deficits.

Although we consider our study an original contribution to the field of neuroaesthetic research, it presents a number of limitations that must be acknowledged.

First, the essentiality of two note intervals limits the range and the intensity of aesthetic responses. On the other hand, the use of richer stimuli would inevitably introduce potential confounds into the results, such as cognitive, perceptual, emotional, situational, socio-cultural, affiliation and historical factors^116^. Nevertheless, future studies should attempt testing the *disinterested interest hypothesis* employing more elaborated stimuli such as complex musical chords (rather than two-note intervals), photos or paintings. Secondly, although the occurrence of attentional enhancement and motor inhibition in the auditory modality are coherent with previous findings from neuroimaging studies^20–22^ investigating aesthetic appreciation across sensory modalities (see Nadal^30^ for a review), the issue of the level of generality of our results across sensory modalities has not been addressed in our study (i.e., modality dependence vs independence). Indeed, it was shown that more complex aesthetic experiences which extend beyond “core liking”, such as the appreciation of works of art (i.e. paintings and music pieces), entail both modality-independent and modality-specific processes^52^. Based on the evidence from previous studies^20–22,30^, we hypothesize that motor inhibition and attentional enhancement emerge during “core liking”^18^ independently from sensory modality, but additional research is needed to test this hypothesis. Thirdly, for technical constraints, our experimental design did not allow to collect AJs and RTs simultaneously while registering EEG activity: this prevented the investigation of the relationship between aesthetic appreciation and motor inhibition on a trial-by-trial basis, which would have increased the internal validity of our study. Lastly, the experimental procedures employed in our tasks differed too much to allow for a direct comparison of the results of the (more contemplative) *AJ* task and the (more pragmatic) *Detection* task. Future studies should specifically address the issue of the effect of the nature of the task (e.g. aesthetic judgement vs. pragmatic judgement) on motor and attentional responses to more appreciated stimuli.

## Methods

### Participants

Forty-four right-handed healthy volunteers participated to the study. Twenty-two participants (12 females; age: 24.45 ± 1.96; years of education: 16.45 ± 1.36) took part to *Experiment 1* (*AJ* task) and *2* (*Detection* task). The remaining twenty-two (13 females; age: 25.75 ± 2.11; years of education: 16.71 ± 1.72) participated in *Experiment 3* (*Go-NoGo* task). The order of Experiments 1 and 2 was counterbalanced among subjects: half of the participants started with *Experiment 1*, while the remaining half started with *Experiment 2*. All participants gave their written informed consent to participate to the study. The study conformed to the standards required by the Declaration of Helsinki and was approved by the local ethics committee (University of Turin).

### Stimuli

Musical Intervals were created with *Csound* (https://csound.com/) software, which allowed to specify the frequency (Hz) of single notes composing the interval. Different types of two-note intervals were defined by the ratio between the frequency of the two notes. Although not exclusively^117,118^, consonance also depends on this ratio: the smaller the numbers that define the ratio, the more consonant will be the resulting interval^36^. Octaves (consonant) were composed by notes with a ratio of 2:1, fifth intervals (mildly dissonant) had a ratio of 3:2, while tritons (dissonant) were defined by a ratio of 45:32. We created seven intervals for each ratio type by varying the frequency of the first note from 200 Hz to 260 Hz (middle C) by steps of 10 Hz. The second note varied according to the ratio described above. In Table 3 we report the frequency of the notes of all the intervals we employed.

**Table 3:** Stimuli.

Frequency of the first note (Hz)	Frequency of the second note (Hz)		
	Octave	Fifth	Tritone
200	100	133.33	142.22
210	105	140	149.33
220	110	146.66	156.44
230	115	153.33	163.55
240	120	160	170.66
250	125	166.66	177.77
260	130	173.33	184.88

First and second notes were displayed simultaneously for 50 ms. Seven intervals with varying frequencies were displayed for each interval type.

In the *Detection* (Experiment 2) and in the *Go-NoGo* tasks (Experiment 3) intervals were intermixed with randomly generated white noise sounds (some trials contained intervals, others contained white noise). Both intervals and white noise were displayed via loudspeakers at the same output intensity (65 dB) for 50 ms. We chose short presentation durations to limit as much as possible the potentially detrimental effect of stimuli offset on EEG signals, given the fact that offset responses are inversely proportional to the duration of the prior sound^119^.

### Apparatus

The set up was identical in the three experiments. Participants sat at a table in a fixed position, distant 60 cm from the loudspeakers and from a 53 cm (diagonal) computer screen, with the screen centre and loudspeakers placed one next to the other and aligned with the trunk midline. The participant’s left arm was resting on the corresponding leg, while the right arm was placed on the desk. Subjects had their index finger resting on the keyboard spacebar during the *Detection* and the *Go-NoGo* tasks. Response keys and subjects’ right hand were aligned with the trunk vertical axis. AEPs were registered during all experiments.

### Experimental procedures

#### Experiment 1 (AJ task)

**Figure 5:**
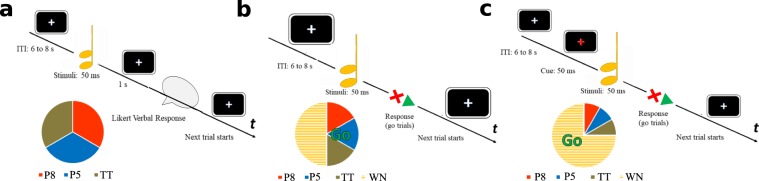
Trial timeline. Panel a shows the trial timeline for the *AJ* task: after the two-note interval was played participants remained still for one second and then verbally reported their answer. Panels b and c show the single-trial timeline of the *Detection* and *Go-NoGo* tasks, respectively: participants were instructed to press the spacebar only when hearing intervals in the *Detection* task. Contrarily they had to respond only when hearing white noise in the *Go-NoGo* task. Sounds were preceded (1 s) by a visual cue (fixation cross turning red for 50 ms) in the *Go-NoGo* task. Pie charts represent the proportion between perfect octave intervals (P8), perfect fifth intervals (P5), tritone intervals (TT) and white noise (WN) in each experiment.

*Experiment 1* consisted of two identical runs. In each run participants were asked to evaluate the beauty of musical intervals using a Likert scale ranging from 1 to 9 (1 = Most ugly, 9 = Most beautiful). Each of the 21 intervals we created was evaluated twice in each of the two runs (for a total of 28 judgements for each interval type in the whole experiment). The trial timeline is depicted in Fig. 5. Intervals were presented in a random order for 50 ms after a variable inter-trial interval (range: 6–8 s). Participants fixated a central white cross for the whole experiment. When they heard an interval, they were asked to wait 1 second until the cross changed into a question mark and then verbally report their evaluation. AJs were recorded by the experimenter using a keyboard and were automatically registered by E-Prime 2.0 software (Psychology Software Tools, Inc. USA). Participants had a five minutes break between runs. Each run lasted approximately 8 minutes.

#### Experiment 2 (Detection task)

*Experiment 2* consisted of two runs of a simple detection task employing the same musical intervals of the *AJ* task. Intervals were intermixed with 50 ms of white noise. Each of the 21 intervals we created was presented twice in each of the two runs (for a total of 28 presentations for each interval type in the whole experiment). The white noise was presented 42 times in each run (white noise and interval trials were equally numerous). The trial timeline is depicted in Fig. 5. Intervals were presented in a random order after a variable inter-trial interval ranging from 6 to 8 s. Participants fixated a central white cross for the whole experiment. They were instructed to press the spacebar as fast as possible as soon as they heard an interval and to restrain from responding when they heard a white noise. Response time (RT) and response accuracy were automatically registered by the experimental software.

#### Experiment 3 (Go-NoGo task)

*Experiment 3* consisted in a *Go-NoGo* task similar to *Experiment 2* except that: 1) subjects had to respond to the white noise and refrain from responding when they heard an interval; 2) the fixation cross turned red for 50 ms (preparatory cue) 1 s before the sound (Go-Nogo signal) was played; 3) white noise and intervals were not equally numerous. Intervals (No-Go trials) were rarer than white noise (Go trials), with a proportion of one to three (28 intervals per interval type and 252 white noises for a total of 336 trials). In each run the 21 intervals were presented twice, randomly alternated with 126 white noise sounds. Additionally, after the *Go-NoGo* task participants of *Experiment 3* performed a brief *AJ* task identical to *Experiment 1* described above but with shorter ITI (2–3 s). AEPs were not registered during this second phase. The Go-NoGo task was devised to elicit those ERP components that are usually associated to the motoric stages of response inhibition (P3) during the presentation of intervals (*NoGo* stimuli), under the assumption that more appreciated intervals should facilitate the inhibition of motor response, therefore amplifying motor-inhibition-related components.

### Electrophysiological recordings and preprocessing

EEG activity was recorded by 32 Ag-AgCl electrodes placed on the scalp of the participant according to the International 10–20 system and referenced to the nose. Electrode impedances were kept below 5 kΩ. The electro-oculogram (EOG) was recorded from two surface electrodes placed over the right lower eyelid and lateral to the outer canthus of the right eye. Signals were recorded and digitized by using a *HandyEGG* (Micromed, Treviso – IT) amplifier with a sampling rate of 1024 Hz.

EEG data were pre-processed and analysed with Letswave6 toolbox (Nocions, Ucl. BE) for Matlab (Mathworks, Inc. USA). Continuous EEG data were divided into epochs of 1.5 s (total duration), including 500 ms pre-stimulus and 1 s post-stimulus intervals. Epochs were band-pass filtered (1–30 Hz in *Experiment 1* and *2*) using a fast Fourier transform filter. In *Experiment 3* epochs were band-pass filtered with a broader filter (0.5–30 Hz) in order to better evidence later components (expressed in lower frequencies), such as P300, that usually emerge in Go-Nogo tasks^51^. Filtered ERPs were baseline corrected using the interval from −0.5 to 0 s as a baseline. Artefacts due to eye movements were eliminated using Independent Component Analysis (ICA^120^). Epochs belonging to the same interval type were then averaged, to obtain three average waveforms (i.e. Octaves, Fifths, Tritones) for each subject. In the *Go-NoGo* task (*Experiment 3*) we additionally analysed epochs corresponding to Go-trials (white noise trials) which were averaged together.

### Data analysis

#### Behavioural data

Outliers (RTs diverging more than 2.5 standard deviations from each single subject’s average value) in the *Detection* task (*Experiment 2*) were excluded from subsequent analyses^121,122^. AJs from *Experiment 1* and outlier-corrected RTs from *Experiment 2* were then averaged across trials with the same interval (each interval was presented 4 times in both experiments; *§ 2.4- Aesthetic judgement task*) to obtain 21 average values per participant.

Single subjects’ averaged RTs were entered as a dependent variable in a linear mixed-model with subjects’ ID as a random-effect factor and AJs as a covariate (fixed-effect factor). This analysis was based on 462 observations (21 per each of the 22 participants).

In *Experiment 3*, omission error rates in the *Detection* task (i.e. incorrect Go trials) and commission error rates in the *Go-NoGo* task (i.e. incorrect NoGo trials) were computed for each interval type for each participant. Single subjects’ AJs from *Experiment 3* were averaged across interval types to obtain three average values per participant.

#### EEG data

First, we were interested in identifying the waveform components modulated by interval type. To test for significant differences among AEP elicited by different interval types, we performed three (one per each experiment) one-way, repeated measures, point-by-point ANOVA^123,124^, with three levels corresponding to the three interval types. Correction for multiple comparisons was applied via clustersize-based permutation testing^125^ (1000 permutations; alpha level = 0.05; percentile of mean cluster sum = 95). Significant clusters were based on both temporal contiguity and spatial adjacency of a minimum of two electrodes.

Furthermore, to investigate the relation between EEG responses and AJs more directly and to further explore the reliability of the point-by-point ANOVA results, we computed a point-by-point trial-by-trial (i.e. considering each single epoch for each single subject separately) correlation analysis^126^ between the amplitude of the EEG responses from single trials (N = 84) registered during the *AJ task* and the corresponding AJ (*§ 2.4- Aesthetic judgement task*). The outcome of the correlation analysis was a 1.5 s (from 0.5 s pre-onset to 1 s post-onset) long time series of r-values for each channel for each subject. This constituted the input for a group-level two-tailed point-by-point t-test with permutation-based correction for multiple comparisons (1000 permutations; alpha level = 0.05; percentile of mean cluster sum = 95; minimum number of adjacent channels = 2). The test compared single subjects’ correlation coefficients against the constant 0 at each time point. This allowed to identify time-clusters containing signal amplitudes which significantly correlated with AJs.

To further test the presence of a possible enhancement in attention- and motor inhibition-related AEP components for more appreciated intervals, in *Experiment 2* and *3*, where it was not possible to compute point-by-point trial-by-trial correlations between AJs and voltages (since EEG responses and AJs were not simultaneously collected in *Experiments* 2 and 3), we extracted single-subjects’ peak amplitudes from relevant waveform components (N1, P2 and N2 in *Experiment 2*; N1 and P3 in *Experiment 3*). Peaks were extracted from single subjects’ average AEP corresponding to the three different interval types. Peaks were defined as the lowest or highest amplitude (for negative and positive components respectively) registered within significant time-cluster evidenced by the point-by-point ANOVA. For each component separately, we performed a one-way repeated measure ANOVA employing peak amplitude as dependent variable and interval type as a within-subject factor (3 levels: Octave, Fifths, Tritones).

Single subjects’ AEPs and correlations between trial-by-trial amplitudes and AJs are available at Mendeley.com.

## Data Availability

Data are available at Mendeley.
